# Tunable Negative Poisson's Ratio in Van der Waals Superlattice

**DOI:** 10.34133/2021/1904839

**Published:** 2021-04-10

**Authors:** Xiaowen Li, Xiaobin Qiang, Zhenhao Gong, Yubo Zhang, Penglai Gong, Lang Chen

**Affiliations:** Department of Physics, Southern University of Science and Technology, Shenzhen, Guangdong 518055, China

## Abstract

Negative Poisson's ratio (NPR) materials are functional and mechanical metamaterials that shrink (expand) longitudinally after being compressed (stretched) laterally. By using first-principles calculations, we found that Poisson's ratio can be tuned from near zero to negative by different stacking modes in van der Waals (vdW) graphene/hexagonal boron nitride (G/*h*-BN) superlattice. We attribute the NPR effect to the interaction of *p*_*z*_ orbitals between the interfacial layers. Furthermore, a parameter calculated by analyzing the electronic band structure, namely, distance-dependent hopping integral, is used to describe the intensity of this interaction. We believe that this mechanism is not only applicable to G/*h*-BN superlattice but can also explain and predict the NPR effect in other vdW layered superlattices. Therefore, the NPR phenomenon, which was relatively rare in 3D and 2D materials, can be realized in the vdW superlattices by different stacking orders. The combinations of tunable NPRs with the excellent electrical/optical properties of 2D vdW superlattices will pave a novel avenue to a wide range of multifunctional applications.

## 1. Introduction

Negative Poisson's ratio (NPR) material shrinks laterally when axially compressed or laterally expands when subjected to axial stretching. Compared to positive Poisson's ratio (PPR) materials, NPR material has higher indentation resistance [1], larger impact resistance, more superior sound absorption performance, and better crack propagation resistance [2]. In addition, when subjected to out-of-plane bending moments, the NPR material will exhibit a dome shape rather than the PPR material tending to saddle shape. These excellent properties indicate that the NPR materials have broad application prospects in the automotive, aerospace, marine, and other industrial fields [3].

The NPR phenomena have been found in many natural and artificial materials, such as cubic metals [4], porous polymers [5], honeycombs [6], perovskite [7, 8], silicon oxides [7, 8], ceramic aerogels [9], reentrant crystal structures [10–17], and liquid crystal elastomer [18]. Recently, the NPR effect has also been found in some two-dimensional (2D) materials [19], such as black phosphorus [20, 21], *δ*-phosphorene [22], borophene [23, 24], graphene [25], *h*-BN [26], 1 T-type transition metal dichalcogenides [27], group-IV monochalcogenides [28], Be_5_C_2_ [29], silicon dioxide [30, 31], FeB_6_ [32], B_4_N [33], and Ag_2_S [34]. In addition, there are hundreds of thousands of materials in the inorganic crystal structure database (ICSD); however, the number of NPR materials reported in the study is a few hundred, which is relatively small. Among them, artificial materials and structures often have a very high NPR, while crystal materials have a relatively small NPR. Especially in 2D materials, NPR is smaller. In 2014, NPR was first found in 2D materials, namely, black phosphorus, with a value of -0.027 [20]. After that, the NPR of the 2D materials found in the later study mostly remains near -0.1. Based on our theoretical calculations, unlike pure 2D materials, the NPR in the vdW heterostructure can only be maintained around -0.1, which is due to the expansion amplitude of *p*_*z*_ orbital under in-plane strain.

Moreover, although many in-depth studies have examined the possible existence of NPR effects in 3D and 2D materials, few studies have reported the NPR effect in vdW superlattice. With the development of manufacturing technology, graphene-based superlattices showed enhanced stability in experiments [35]. Therefore, the designability and diversity of vdW superlattices provide a broad prospect for designing multifunctional NPR materials, such as NPR electrodes and molecular sieves [36]. In previous studies, the most NPR phenomena can be attributed to its unique wrinkle or re-entrant structures. In addition to these special geometric reasons, we want to study the fundamental physical mechanisms that form NPR effect.

In our previous study, we reported near-zero Poisson's ratio (ZPR) phenomena in G/*h*-BN and multilayer *h*-BN [37]. Interestingly, in this study, using first-principles calculations, we found that Poisson's ratios of G/*h*-BN superlattices are -0.109, -0.111, and 0.023 in different stacking modes. The dichotomy between NPR and PPR effects exhibited in the G/*h*-BN superlattice, which can be explained by a special electronic structure at the interfacial layer. Although G/*h*-BN is a kind of simple vdW heterostructure, it is convenient to make theoretical analysis and calculation clearly. It may open a beginning for the theoretical study of NPR effect in different stacking modes of vdW materials. In addition, we calculated the out-of-plane stiffness of the G/*h*-BN superlattice with different stacking modes. These modes with NPR also have out-of-plane negative shear modulus (NSM), i.e., when shear strain is applied to NSM materials, as the shear strain increases, the corresponding shear stress tends to decrease.

Ultimately, the NPR phenomenon, which was relatively rare in 3D and 2D materials, can be realized in the vdW superlattices by different stacking orders as designed. Furthermore, studying on how to change the PPR material into the NPR material not only has important practical engineering application value but also theoretical value for in-depth study on other possible related interesting physical properties, such as negative pressure electricity, negative stiffness, and negative thermal expansion.

## 2. Results

The unit cell of the G/*h*-BN superlattice is composed of 1 × 1 graphene unit cell (2 C atoms) and 1 × 1 *h*-BN unit cell (1 B atom and 1 N atom) in the *x*-*y* plane. The lattice constant *a* of the pristine monolayer graphene and *h*-BN are 2.465 Å and 2.509 Å, respectively. Then, the *a* of G/*h*-BN superlattice calculated by first-principles calculations is 2.485 Å, and the lattice mismatch between graphene and *h*-BN is less than 1%.

The interlayer binding energies (*E*_bind_) and equilibrium distances (*d*) of all stacking modes of G/*h*-BN superlattices obtained by density functional theory (DFT) approach are given in Table S1. Here, we investigated three highly symmetric stacking modes of G/*h*-BN superlattices: N atom sublattice on hexagonal C atom ring (stacking mode A), B atom sublattice on C atom ring center (stacking mode B), and N atom sublattice on C atom ring center (stacking mode C) [38]. *E*_bind_ follows the order of *E*_bind (A)_ < *E*_bind (B)_ < *E*_bind (E)_, while *d* follows the order of *d*_A_ > *d*_B_ > *d*_C_.

### 2.1. Stiffness

By analyzing the strain energy, the elastic constants of G/*h*-BN superlattices were derived from the linear fitting of the energy-strain relationship (Table S2). For hexagonal crystal, the in-plane mechanical properties of G/*h*-BN superlattice are isotropic (*Y*_11_ = *Y*_22_, *v*_12_ = *v*_21_, *v*_13_ = *v*_23_) [41]. Young's modulus *Y*_*αα*_ is determined by elastic constants *C*_*αβ*_ (see method section). Notably, the in-plane *Y*_11_ of the 2D material is the product of the *Y*_11_ of the corresponding 3D material and the effective thickness [27], and we took the *d* as the effective thickness for each layer of 2D material.

Table 1 shows that the out-of-plane *Y*_33_ of the stacking mode A, B, and C is 44.9, 45.6, and 49.0 GPa, respectively. The smaller the *d* of the stacking mode, the greater the corresponding *Y*_33_. In addition, we calculated the in-plane *Y*_11_ of the G/*h*-BN superlattice, which is almost equal to the sum of *Y*_11_ of the monolayer graphene and *h*-BN. Therefore, this result explains the reason for the stability enhancement of carbon-based superlattices observed in experiments [35]. However, the difference in *Y*_11_ of the superlattice is mainly due to the different in *d*.

### 2.2. Poisson's Ratio

**Figure 1:**
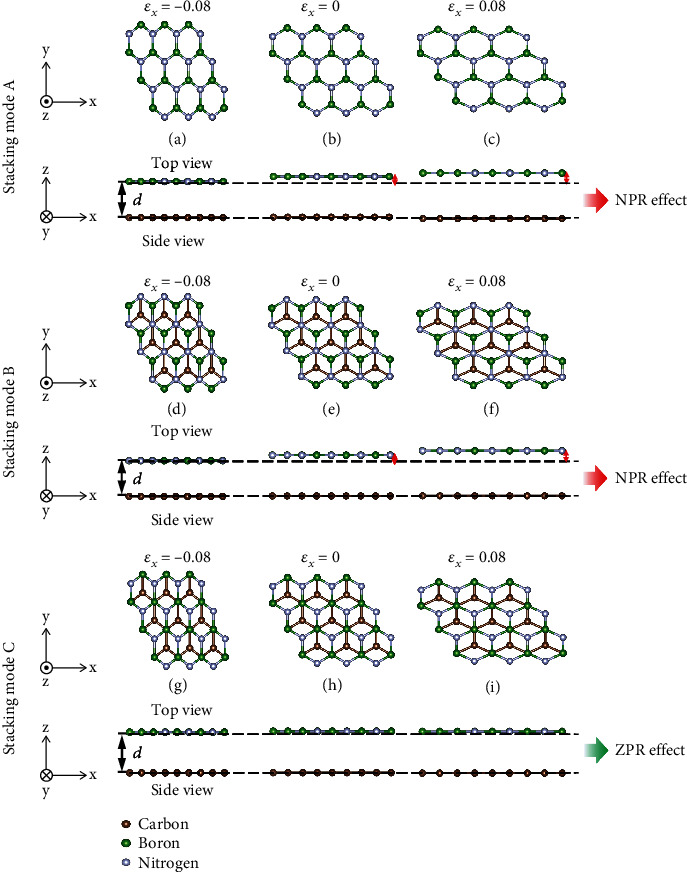
Top and side views of G/*h*-BN superlattices in different stacking modes under in-plane strain (a, d, g) *ε*_*x*_ = −0.08, (b, e, h) *ε*_*x*_ = 0, and (c, f, i) *ε*_*x*_ = 0.08. Here, *d* represents interfacial layer equilibrium distance.

We compared G/*h*-BN superlattices in different stacking modes under different uniaxial strains along the *x* direction (*ε*_*x*_) (Figure 1). For stacking modes A and B, the *d* is auxetic for *ε*_*x*_ > 0, but the same phenomenon was not found in stacking mode C. Interestingly, stacking modes A and B not only have the NPR (*v*_13_) effect but they also have negative shear modulus (NSM) (*G*_44_) in the out-of-plane direction. The shear force decreases with the increase of shear deformation, which is the NSM effect.

**Figure 2:**
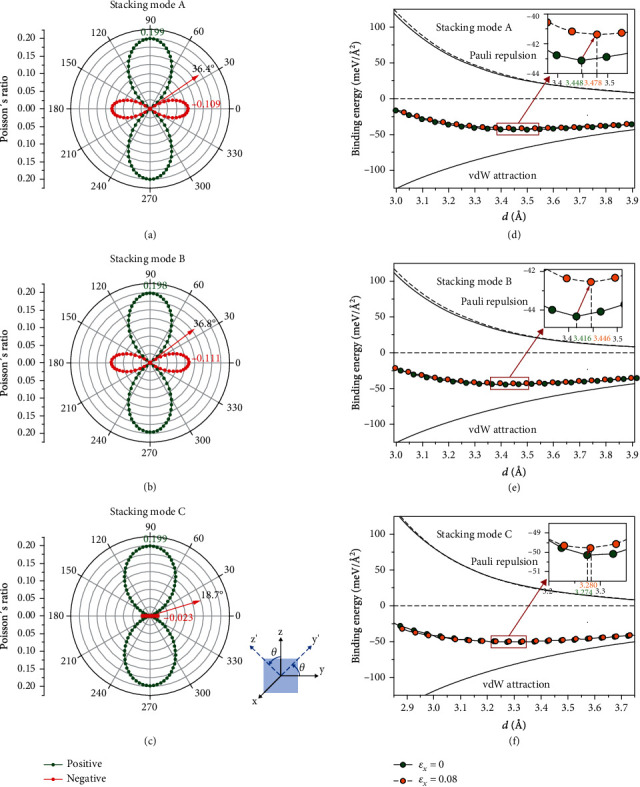
Poisson's ratio *v*_*ij*_(*θ*) (*v*_13_) as a function of G/*h*-BN superlattices in (a) stacking mode A, (b) stacking mode B, and (c) stacking mode C for *i* fixed in the *x* direction and *j* varying in the *y-z* plane. Interlayer binding energy (*E*_bind_) of G/*h*-BN superlattices in (d) stacking mode A, (e) stacking mode B, and (f) stacking mode C with *d*. The fitting energy curves below the horizontal coordinate axis represent vdW attraction, and the fitting energy curves above the horizontal coordinate axis represent Pauli repulsion. These dashed lines represent the fitting energy curves for *ε*_*x*_ = 0.08, and the solid lines represent the fitting energy curves at the equilibrium position. The insets show enlarged energy curves.

In order to study the anisotropy of Poisson's ratio of these materials, orientation-dependent Poisson's ratio was calculated (Figure 2). We found that the stacking modes A, B, and C have the ZPR (*v*_13_) effect at *θ* = 36.4°, 36.8°, and 18.7°, respectively. Therefore, stacking modes A and B exhibit a NPR effect in a larger crystal orientation angle range than stacking mode C.

To our knowledge, it is very difficult to measure Poisson' s ratio of several layers of two-dimensional (2D) material with the existing experimental method. Because for these ultra-thin films, when the in-plane strain is applied, the out-of-plane deformation is very small and difficult to observe. However, for multilayer 2D materials, X-ray diffraction can be easily used to measure Poisson' s ratio when the thickness is close to 10 nm [42]. The NPR effect is generated in the interfacial layer. Therefore, both multi and single-layer vdW materials can exhibit the same NPR. It is relatively easy to measure Poisson' s ratio for the multilayer vdW materials with a certain thickness.

## 3. Discussion

### 3.1. Interlayer Binding Energy

Assuming that the interaction between the two layers of the superlattice is additive, the binding potential can be expressed as the cumulative interaction of atoms between different layers [43]. The binding energy of two atoms combined by vdW forces can be expressed by the Lennard-Jones potential:

(1) $$\begin{array}{c} E\left(r\right)=4\varepsilon \left[-{\left(\frac{\sigma }{r}\right)}^{6}+{\left(\frac{\sigma }{r}\right)}^{12}\right]. \end{array}$$

Here, *r* represents the distance between the two atoms. The *ε* and *σ* are fitting constants. The first term represents the vdW attraction, and the second term represents Pauli's repulsion [44]. Therefore, the interlayer potential of the vdW superlattice can be expressed as

(2) $$\begin{array}{c} \begin{array}{c} E_{\text{bind}}\left(d\right)=\rho_{1}\rho_{2}\int_{0}^{\infty }2\pi xE\left(r\right)dx=2\pi \varepsilon \rho_{1}\rho_{2}\left(-\frac{\sigma^{6}}{d^{4}}+\frac{2\sigma^{12}}{5d^{10}}\right), \end{array} \end{array}$$

where *ρ*_1_ and *ρ*_2_ are the mass densities of two layers of vdW superlattice, respectively. The distance $r\left(x\right)=\sqrt{x^{2}+d^{2}}$ is obtained from the geometric relationship between the coordinate *x* and *d*. For G/*h*-BN superlattice, *ρ*_1_ = *ρ*_2_. In Figure 2, the fitting curves of ~ *d*^−4^ below the horizontal coordinate axis represent vdW attraction, and the curves of ~ *d*^−10^ above the horizontal coordinate axis represent Pauli repulsion. Therefore, the low-order vdW term plays a major role in the large *d*, while high-order Pauli's repulsion term plays a major role in the small *d*. According to the first-principles calculation, Eq. (2) can well describe the *E*_bind_ of the vdW superlattice.

Figures 2(d) and 2(e) show that when *ε*_*x*_ = 0.08, the Pauli repulsion energy increases significantly, while the vdW attraction energy has a negligible change. Therefore, with the decrease of *E*_bind_, *d* of the G/*h*-BN superlattice expands, while the lowest point of the energy curve moves forward along the positive direction of the *x* axis, resulting in a negative *v*_13_ of -0.109 and -0.111. For the stacking mode C, vdW and Pauli repulsion show negligible changes under *ε*_*x*_ = 0.08 (see Figure 2(f)). The binding energy increases slightly; so, the G/*h*-BN superlattice exhibits the ZPR effect. According to the first-principles calculation, Poisson's ratio of the material has a relationship with *σ*. If the material can exhibit the NPR effect, the value of *σ* under tensile strain (*σ*′) is greater than the initial value of *σ* (Table S4). However, we calculated that the increase of *ε* is not a necessary condition for the NPR effect, i.e., *σ* plays a major role in the NPR effect.

### 3.2. Relationship between *p*_*z*_ Orbitals and NPR

The Bloch wave function of the *p*_*z*_ orbital electron in a periodic lattice under the tight binding (TB) approximation can be expressed as

(3) $$\begin{array}{c} \begin{array}{c} \phi_{A\left(B\right)}\left(\overset{⃑}{k},\overset{⃑}{r}\right)=\frac{1}{\sqrt{N}}\underset{m}{\sum }e^{i\overset{⃑}{k}\cdot {\overset{⃑}{R}}_{m}}\varphi_{A\left(B\right)}\left(\overset{⃑}{r}-{\overset{⃑}{R}}_{m}\right). \end{array} \end{array}$$

**Figure 3:**
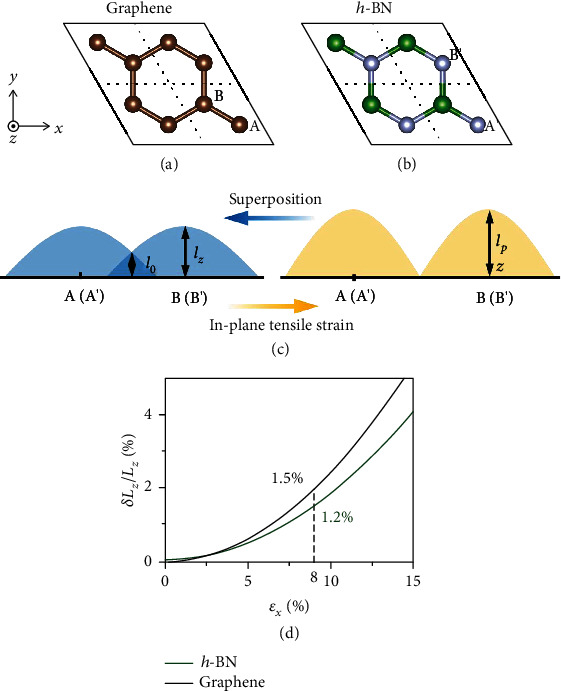
Unit cell of (a) graphene and (b) *h*-BN. (c) Wave function superposition of A and B atoms. (d) The length of *p*_*z*_ orbital in each layer of G/*h*-BN superlattice under different strains. Here, *a* represents the distance between A and B atoms. Under the in-plane strain *ε*_*x*_ = *δ*_*x*_/*a*, the length of *p*_*z*_ orbital is *L*_*Z*_ + *δL*_*Z*_.

For G/*h*-BN superlattice, C atoms in graphene and N atoms in *h*-BN have *p*_*z*_ orbitals. When the atom A is used as the origin of coordinates (Figures 3(a) and 3(b)), let the in-plane strain be the perturbation $\overset{⃑}{\delta }$. The wave function of *p*_*z*_ orbitals of the atom A is $\phi_{A}\left(\overset{⃑}{k},\overset{⃑}{r}\right)$. Meanwhile, the wave function of *p*_*z*_ orbitals of the atom B is $\phi_{B}\left(\overset{⃑}{k},\overset{⃑}{r}-\overset{⃑}{\delta }\right)$. When the A and B atoms in the lattice are bonded, the wave function of the bonded *p_z_* orbital can be expressed by the linear combination of atomic orbitals as

(4) $$\begin{array}{c} \begin{array}{c} \psi_{p_{z}}\left(\overset{⃑}{k},\overset{⃑}{r}\right)=C\left(\phi_{A}\left(\overset{⃑}{k},\overset{⃑}{r}\right)+e^{-i\overset{⃑}{k}\cdot {\overset{⃑}{\delta }}_{j}}\phi_{B}\left(\overset{⃑}{k},\overset{⃑}{r}\right)\right). \end{array} \end{array}$$

The constant *C* is a normalization constant, which should satisfy the normalization condition $\langle \psi_{p_{z}}\left(\overset{⃑}{k}\right)|\psi_{p_{z}}\left(\overset{⃑}{k}\right)\rangle =1$. The density-weighted length of the *p*_*z*_ electrons in the out-of-plane direction can be expressed as

(5) $$\begin{array}{c} \begin{array}{c} l_{z}\left(\overset{⃑}{k}\right)=\langle \psi_{p_{z}}\left(\overset{⃑}{k}\right)\;|\;\left|z\right|\;|\;\psi_{p_{z}}\left(\overset{⃑}{k}\right)\rangle . \end{array} \end{array}$$

Here, $l_{z}\left(\overset{⃑}{k}\right)$ is the length of the *p*_*z*_ electrons with momentum $\overset{⃑}{k}$ in the out-of-plane direction. Therefore, the length (*L*_*z*_) of *p*_*z*_ electrons with all momentum should be the integral of $l_{z}\left(\overset{⃑}{k}\right)$ in the first Brillouin zone (BZ). Finally, the charge density-weighted length of the *p*_*z*_ orbital in the out-of-plane direction can be obtained:

(6) $$\begin{array}{c} \begin{array}{c} L_{z}\left(\overset{⃑}{\delta }\right)=\frac{1}{S_{BZ}}\iint_{S_{BZ}}l_{z}\left(\overset{⃑}{k}\right){dk}^{2}=l_{p_{z}}f\left(\overset{⃑}{\delta }\right), \end{array} \end{array}$$

where $f\left(\overset{⃑}{\delta }\right)=1+\left(1/3S_{BZ}\right)\iint_{S_{BZ}}\left(2e^{ik_{x}\left(a/2\right)}\cos\left(k_{y}\left(\sqrt{3}a/2\right)\right)+e^{-ik_{x}a}\right)\cos\left(\overset{⃑}{k}\cdot \overset{⃑}{\delta }\right){dk}^{2}$ and $l_{p_{z}}\left(\overset{⃑}{k}\right)=\langle \varphi \left(\overset{⃑}{r}-{\overset{⃑}{R}}_{m}^{A}\right)|\left|z\right||\varphi \left(\overset{⃑}{r}-{\overset{⃑}{R}}_{m}^{A}\right)\rangle$, which is the length of the isolated *p*_*z*_ orbital (Figure 3(c)). Therefore, according to Eq. (6), we got the analytical solution of the relationship between *ε*_*x*_ and $L_{z}\left(\overset{⃑}{\delta }\right)$ (Figure 3(d)). The calculation details can be found in the Supporting Information. Meanwhile, partially differentiate $f\left(\overset{⃑}{\delta }\right)$ to the in-plane perturbation ${\overset{⃑}{\delta }}_{j}$ is $\partial f\left(\overset{⃑}{\delta }\right)/\partial \overset{⃑}{\delta }>0$. Therefore, *p*_*z*_ orbitals will extend out-of-plane under in-plane tensile strain.

Since the length of the *p*_*z*_ orbital has auxetic effect under in-plane strain, we quantitatively studied the charge density distribution along the out-of-plane direction of G/*h*-BN superlattice by using first-principles calculations. In the out-of-plane direction, the charge density at coordinate *z* can be expressed as

(7) $$\begin{array}{c} \begin{array}{c} P_{z}\left(z\right)=\int_{-\infty }^{\varepsilon_{F}}\int \rho_{E}\left(x,y,z\right)dxdydE. \end{array} \end{array}$$

Here, *ρ*_*E*_(*x*, *y*, *z*) is the charge density at the coordinate (*x*, *y*, *z*) with the energy of *E*, and *ε*_*F*_ is the Fermi level of the system. In order to quantify the change of the charge density in the out-of-plane direction under stress, we calculated the weighted length of the electron density in the out-of-plane direction according to the following formula:

(8) $$\begin{array}{c} \begin{array}{c} L_{z}=\frac{\int \left|z\right|P_{Z}\left(z\right)dz}{\int P_{Z}\left(z\right)dz}. \end{array} \end{array}$$

Notably, the charge of the graphene in the out-of-plane direction is mainly contributed by the *p*_*z*_ orbital. The *L*_*z*_ of each layer in the G/*h*-BN superlattice under in-plane strain *ε*_*x*_ = 0 and *ε*_*x*_ = 0.08 is shown in Table S4. In each layer of the G/*h*-BN superlattice, the *L*_*z*_ is elongated. When an in-plane strain *ε*_*x*_ = 0.08 was applied, the bond angle ∠NBN increased from 120° to 122.36°, resulting in the charge localization (Fig. S2).

**Figure 4:**
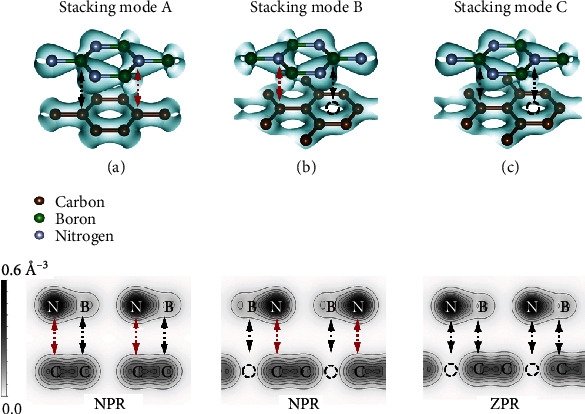
The charge density with the isosurface of 0.103 Å^−3^ and the electron localization function (ELF) with the Miller indices of (1 1 0) of *h*-BN superlattices in (a) stacking mode A, (b) stacking mode B, and (c) stacking mode C. The red dotted arrows and circles indicate the overlap of the *p*_*z*_ orbits, while the black dotted arrows and circles show that the overlap of the *p*_*z*_ orbitals does not actually exist.

Quantitatively, we found that the value of *L*_*Z*_ of the monolayer *h*-BN and graphene in G/*h*-BN superlattices increased by 1.8 ~ 1.9% and 2.3 ~ 2.4%, as the in-plane tensile strain increases by 8% (Table S5), explaining the NPR effect in stacking modes A and B along the out-of-plane direction. This is consistent with the analytical solution obtained by TB approximation (Figure 3(d)). For stacking mode C, the N atom sublattice is on the C atom ring center. In *h*-BN, the N atom has a fully filled *p*_*z*_ orbital, while the B atom has an empty *p*_*z*_ orbital. The *p*_*z*_ orbitals of G/*h-*BN superlattices hardly overlap (Figure 4(c)); so, the change of the *p*_*z*_ orbitals has little effect on the Pauli repulsion between the interfacial layers, resulting in no significant NPR effect.

### 3.3. Relationship between Electronic Band Structures and NPR

**Figure 5:**
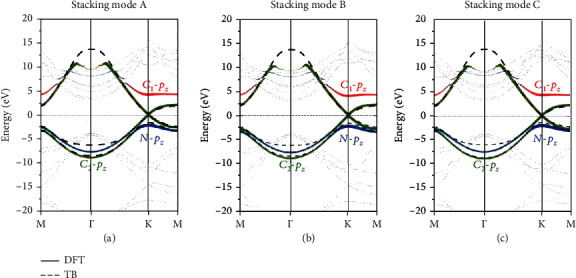
Electronic band structures of G/*h*-BN superlattices in different stacking modes from DFT (gray) and TB (black) calculations. Red, green, and blue denote the contributions of the *p*_*z*_ orbitals of C_1_, C_2_, and N atoms, respectively.

Figure 5 shows the DFT and TB-based band structure of G/*h*-BN superlattices in different stacking modes. To further understand the first-principles calculation results, we adopted the TB model to describe the electrons in G/*h*-BN superlattices with different stacking modes near the Fermi level. During the TB calculation, a unit cell contains two C_1_ and C_2_ carbon atoms at different positions and one N atom. Since the electronic states of the three bands around the Fermi level are completely contributed by the *p*_*z*_ orbitals of C_1_, C_2_, and N atoms, only the *p*_*z*_ orbitals of C_1_, C_2_, and N atoms are included in the TB model. The Hamiltonian matrix can be written as

(9) $$\begin{array}{c} \begin{array}{c} \left(\begin{array}{ccc} H_{11} & H_{12} & H_{13}\\ {H_{12}}^{*} & H_{22} & H_{23}\\ {H_{13}}^{*} & {H_{23}}^{*} & H_{33} \end{array}\right), \end{array} \end{array}$$

where subscripts 1, 2, and 3 represent C_1_, C_2_, and N atoms, respectively. Because the interlayer distance is longer than the C-C bond length, the nearest-neighbor interaction between C and N atoms and the next nearest-neighbor interaction between C and C atoms are considered (detailed Hamiltonian matrix elements can refer to the Supporting Information). The distance-dependent hopping integral is determined by the formula

(10) $$\begin{array}{c} f_{ij\sigma }\left(d_{ij}\right)=V_{ij\sigma }e^{q_{ij}\left(1-\frac{d_{ij}}{d_{0}}\right)}. \end{array}$$

Here, *d*_0_ represents the interfacial layer equilibrium distance, and *V*_*ijσ*_ is the hopping integral between *p*_*z*_ orbitals at *d*_0_. *d*_*ij*_ is the distance between the *i*th and *j*th atoms, and *q*_*ij*_ is the decay constant for the integral [45]. For the G/*h*-BN superlattices, the values of *V*_*ijσ*_ and *q*_*ij*_ can refer to the Supporting Information Table S6.

The distance-dependent hopping integral (*f*_*CNσ*_) describes the intensity of the interaction between the *p*_*z*_ orbitals of C and N atoms. Therefore, *f*_*CNσ*_ is a power-exponential function of the interlayer spacing and is proportional to the NPR (Table 2). Furthermore, the higher the value of *f*_*CNσ*_, the greater the value of the corresponding NPR. Note that after Taylor expansion of the *f*_*CNσ*_(*d*_*ij*_), the quadratic term is the previous research results [46, 47].

Consequently, the vdW superlattice can exhibit an NPR effect only if they have *p*_*z*_ orbitals in the out-of-plane direction, and the *p*_*z*_ orbitals overlap between the interfacial layers. Meanwhile, the NPR effect in all vdW materials can be explained by the same physical mechanism given in this section. For example, for lattice-matched materials, a previous study showed that *AA*-stacked *h*-BN (a N atom on a N atom in another layer) can exhibit an NPR effect [26] (Table 3). In addition, for lattice-mismatched vdW materials, the *p*_*z*_ orbitals between the interfacial layers overlap; so, these materials should exhibit an NPR effect. For example, it has been observed that WS_2_/WSe_2_ heterostructure expands abnormally under engineering tensile strain [48]. Therefore, according to this physical mechanism, the NPR phenomenon should exist in a large number of vdW materials, which was considered as a rare phenomenon in bulk and monolayer 2D materials.

Moreover, in the experiment, the isolated atomic layers can also be reassembled into the designed heterostructure layer by layer in a precisely selected order [49]. Therefore, for the same kind of investigated material, it can also be switched in different stacking modes through experimental methods. For example, the G/*h*-BN superlattices may also be tuned among stacking modes A, B, and C. Similarly, we can change the material without strong interlayer *p*_*z*_ orbital interaction into a material with *p*_*z*_ orbitals strongly overlapping between the interfacial layers, thus exhibiting an NPR effect.

In conclusion, we studied Poisson's ratios and the binding energies of G/*h*-BN superlattices in different stacking modes by using the first-principles method. We found that the stacking mode C has a ZPR effect at the interfacial layer, while the stacking modes A and B show NPR effects. The NPR effect is mainly due to the interaction of the *p*_*z*_ orbitals between the interfacial layers. Furthermore, the distance-dependent hopping integral (*f*_*CNσ*_) calculated by analyzing that the electronic band structure can be used to describe the intensity of this interaction. The *f*_*CNσ*_ is a power-exponential function of the interlayer spacing and is proportional to the NPR. Moreover, we calculated their Young's and shear modulus and found that the stacking modes A and B also have NSM effect in the out-of-plane direction. These materials with negative index coexistence will provide broad prospects for multifunctional and multipurpose materials. Finally, we expect that the theory can be verified by experiments and provide a solid foundation for the large-scale searching and predicting NPR materials in the future.

## 4. Methods

Based on density of functional theory, all first-principles calculations were implemented by the planewave projector augmented wave (PAW) method in Vienna ab initio simulation package (VASP) code [50]. The exchange correlation functional adopted the generalized gradient approximation (GGA) of the Perdew−Burke−Ernzerhof (PBE) functional [51]. In order to test the robustness of our results, the vdW-corrected functionals proposed by Grimme DFT + D2 [52], DFT + D3 [53], many-body dispersion (MBD) [54], and vdW-corrected functional optB88-vdW [55] methods were used in first-principles calculations. In this paper, the calculation results of functional optB88-vdW are given because of its good agreement with the experimental results, and the results obtained by different vdW-corrected methods are only slightly different in numerical value (the detailed results are in the Supporting Information).

The G/*h*-BN superlattice was calculated by using 28 × 28 × 10 Monkhorst–Pack K-point mesh. The energy cut-off value is 500 eV, and the structures were completely relaxed until their atomic Hellmann–Feynman forces were less than 0.005 eV/Å. The convergence criterion of energy in the self-consistency process is 10^−6^ eV. We also calculated electronic band structures for G/*h*-BN superlattices by using the HSE06 hybrid functional [56].

To quantitatively characterize the mechanical properties of the interface, the interlayer binding energy (*E*_bind_) between the monolayer graphene and *h*-BN is as follows:

(11) $$\begin{array}{c} E_{\text{bind}}=\frac{\left|E_{\text{G}/\text{h}-\text{BN}}-\left(E_{\text{G}}+E_{\text{h}-\text{BN}}\right)\right|}{S}, \end{array}$$

where *E*_G/*h*−BN_, *E*_G_, and *E*_*h*−BN_ are the energies of the G/*h*-BN superlattice, graphene, and *h*-BN, respectively. *S* represents the in-plane area of the superlattice.

The elastic constant is defined by expanding the internal energy *E* into Taylor series in elastic strain at constant entropy. The expansion coefficient in the Taylor series is the elastic constant [57]:

(12) $$\begin{array}{c} \begin{array}{c} C_{ijkl}=\rho_{0}{\left.\frac{\partial^{2}E}{\partial \eta_{ij}\partial \eta_{kl}}\right|}_{\eta =0}, \end{array} \end{array}$$

where *ρ*_0_ and *η*_*ij*_ are the initial mass density and the Lagrangian strains of the material [58]. In this work, we use contracted notations (11 → 1, 22 → 2, 33 → 3, 13 → 4, 23 → 5, 12 → 6, *C*_*ijkl*_ → *C*_*αβ*_) as tensor indices. In addition, we define Lagrangian strains *η*_1_ → *ε*_*x*_, *η*_2_ → *ε*_*y*_ and *η*_3_ → *ε*_*z*_. The compliance coefficients *S*_*αβ*_ are defined as

(13) $$\begin{array}{c} \begin{array}{c} \sigma_{\alpha }=\underset{\beta }{\sum }S_{\alpha \beta }\varepsilon_{\beta },\left(\alpha ,\beta =1,2,\cdots ,6\right). \end{array} \end{array}$$

Young's modulus for the material is computed by

(14) $$\begin{array}{c} \begin{array}{c} Y_{\alpha \beta }=\frac{1}{S_{\alpha \beta }}. \end{array} \end{array}$$

Poisson's ratio is defined as

(15) $$\begin{array}{c} \begin{array}{c} v_{ij}=-\frac{\varepsilon_{j}}{\varepsilon_{i}}, \end{array} \end{array}$$

where *ε*_*i*_ is the strain in the direction of uniaxial loading (in the *i*-direction), and *ε*_*j*_ is the resulting strain in the transverse direction (the *j*-direction). In our calculations, we applied different uniaxial strains to the lattice. This strained structure was then completely relaxed to evaluate the magnitude of the strain in the out-of-plane direction. The detailed calculation process of the relationship between *θ* and *ν*_13_ is provided in the Supporting Information.

## Figures and Tables

**Table 1:** Poisson's ratio, Young's, and shear modulus of graphene, *h*-BN and G/*h*-BN.

		Method	In-plane			Out-of-plane			
			*v*_12_	*Y*_11_	*G*_66_	*v*_13_	*v*_31_	*Y*_33_	*G*_44_
G/*h*-BN	Stacking mode A	DFT	0.199	886.8	369.7	-0.109	-0.005	44.9	-1.3
	Stacking mode B	DFT	0.198	896.4	374.2	-0.111	-0.006	45.6	-2.8
	Stacking mode C	DFT	0.199	932.8	389.1	-0.023	-0.001	49.0	8.7

Monolayer	Graphene	Expt. [39]	0.165	340 ± 50	145.9 ± 30				
		DFT	0.159	340	146.7				
	*h*-BN	Expt. [40]	0.19	273	114.7				
		DFT	0.199	238	99.3				

For monolayer materials, the unit of Young's and shear modulus is Nm^−1^. For G/*h*-BN superlattices, the unit of Young's and shear modulus is GPa.

**Table 2:** The values of *d*_0_, *f*_*CNσ*_, and Poisson's ratio of G/*h*-BN superlattices in different stacking modes.

Stacking mode	*d*_0_ (Å)	*f*_*CNσ*_ (eV)	Poisson's ratio
A	3.448	-0.31	-0.109
B	3.416	-0.31	-0.111
C	3.274	-0.22	-0.023

**Table 3:** Poisson's ratios of vdW materials with NPR.

	*v*_13_
Bilayer graphene [26]	-0.09
*AA*-stacked *h*-BN [26]	-0.12
G/MoS_2_ heterostructure [37]	-0.09
G/*h*-BN	
Stacking mode A	-0.109
Stacking mode B	-0.111

## Data Availability

All data needed to evaluate the conclusions in the paper are presented in the paper and supplementary materials. And additional data are available from the corresponding authors upon reasonable request.

## Supplementary Materials

Supplementary Materials 1: interlayer binding energies and equilibrium distances results-Supplementary Table S1 2: elastic constants results-Supplementary Table S2 3: volume modulus and shear modulus results-Supplementary Table S3 4: interlayer binding energy-Supplementary Table S4 5: relationship between *p*_*z*_ orbitals and NPR-Supplementary Figure S1 6: charge density-Supplementary Figure S2, Table S5 7: charge density. Table 8: the relationship between Poisson's ratio and stiffness. The relationship between *θ* and *ν*13 9. The relationship between electronic band structures and NPR-Supplementary Table S6.

## References

[1] Alderson A., Alderson K. L. (2007). Auxetic materials. *Proceedings of the Institution of Mechanical Engineers, Part G: Journal of Aerospace Engineering*.

[2] Yang W., Li Z.-M., Shi W., Xie B. H., Yang M. B. (2004). Review on auxetic materials. *Journal of Materials Science*.

[3] Huang C., Chen L. (2016). Negative Poisson's ratio in modern functional materials. *Advanced Materials*.

[4] Milstein F., Huang K. (1979). Existence of a negative Poisson ratio in fcc crystals. *Physical Review B*.

[5] Caddock B. D., Evans K. E. (1989). Microporous materials with negative Poisson's ratios. I. Microstructure and mechanical properties. *Journal of Physics D Applied Physics*.

[6] Lakes R. (1987). Foam structures with a negative Poisson's ratio. *Science*.

[7] Huang C. W., Ren W., Nguyen V. C. (2012). Abnormal Poisson's ratio and linear compressibility in perovskite materials. *Advanced Materials*.

[8] Yeganeh-Haeri A., Weidner D. J., Parise J. B. (1992). Elasticity of agr-cristobalite: a silicon dioxide with a negative Poisson's ratio. *Science*.

[9] Xu X., Zhang Q., Hao M. (2019). Double-negative-index ceramic aerogels for thermal superinsulation. *Science*.

[10] Babaee S., Shim J., Weaver J. C., Chen E. R., Patel N., Bertoldi K. (2013). 3D soft metamaterials with negative Poisson's ratio. *Advanced Materials*.

[11] Wei Z. Y., Guo Z. V., Dudte L., Liang H. Y., Mahadevan L. (2013). Geometric mechanics of periodic pleated origami. *Physical Review Letters*.

[12] Silverberg J. L., Evans A. A., McLeod L. (2014). Using origami design principles to fold reprogrammable mechanical metamaterials. *Science*.

[13] Ortiz A. U., Boutin A., Fuchs A. H., Coudert F.-X. (2012). Anisotropic elastic properties of flexible metal-organic frameworks: how soft are soft porous crystals?. *Physical Review Letters*.

[14] Hall L. J., Coluci V. R., GalvaO D. S. (2008). Sign change of Poisson's ratio for carbon nanotube sheets. *Science*.

[15] Song F., Zhou J., Xu X., Xu Y., Bai Y. (2008). Effect of a negative Poisson ratio in the tension of ceramics. *Physical Review Letters*.

[16] Williams J. J., Smith C. W., Evans K. E., Lethbridge Z. A. D., Walton R. I. (2007). Off-Axis elastic properties and the effect of extraframework species on structural flexibility of the NAT-type zeolites: simulations of structure and elastic properties. *Chemistry of Materials*.

[17] Pham M.-S., Liu C., Todd I., Lertthanasarn J. (2019). Damage-tolerant architected materials inspired by crystal microstructure. *Nature*.

[18] Mistry D., Connell S. D., Mickthwaite S. L., Morgan P. B., Clamp J. H., Gleeson H. F. (2018). Coincident molecular auxeticity and negative order parameter in a liquid crystal elastomer. *Nature Communications*.

[19] Peng R., Ma Y., Wu Q., Huang B., Dai Y. (2019). Two-dimensional materials with intrinsic auxeticity: progress and perspectives. *Nanoscale*.

[20] Jiang J. W., Park H. S. (2014). Negative poisson's ratio in single-layer black phosphorus. *Nature Communications*.

[21] Du Y., Maassen J., Wu W., Luo Z., Xu X., Ye P. D. (2016). Auxetic black phosphorus: a 2D material with negative Poisson's ratio. *Nano Letters*.

[22] Wang H., Li X., Li P., Yang J. (2017). *δ*-Phosphorene: a two dimensional material with a highly negative Poisson's ratio. *Nanoscale*.

[23] Kou L., Ma Y., Tang C., Sun Z., Du A., Chen C. (2016). Auxetic and ferroelastic borophane: a novel 2D material with negative Possion's ratio and switchable dirac transport channels. *Nano Letters*.

[24] Mannix A. J., Zhou X. F., Kiraly B. (2015). Synthesis of borophenes: anisotropic, two-dimensional boron polymorphs. *Science*.

[25] Zhang Q., Xu X., Lin D. (2016). Hyperbolically patterned 3D graphene metamaterial with negative Poisson's ratio and superelasticity. *Advanced Materials*.

[26] Woo S., Park H. C., Son Y.-W. (2016). Poisson's ratio in layered two-dimensional crystals. *Physical Review B*.

[27] Yu L., Yan Q., Ruzsinszky A. (2017). Negative Poisson's ratio in 1T-type crystalline two-dimensional transition metal dichalcogenides. *Nature Communications*.

[28] Kong X., Deng J., Li L. (2018). Tunable auxetic properties in group-IV monochalcogenide monolayers. *Physical Review B*.

[29] Wang Y., Li F., Li Y., Chen Z. (2016). Semi-metallic Be5C2 monolayer global minimum with quasi-planar pentacoordinate carbons and negative Poisson's ratio. *Nature Communications*.

[30] Gao Z., Dong X., Li N., Ren J. (2017). Novel two-dimensional silicon dioxide with in-plane negative Poisson's ratio. *Nano Letters*.

[31] Özçelik V. O., Cahangirov S., Ciraci S. (2014). Stable single-layer honeycomblike structure of silica. *Physical Review Letters*.

[32] Li J., Wei Y., Fan X. (2016). Global minimum of two-dimensional FeB6and an oxidization induced negative Poisson's ratio: a new stable allotrope. *Journal of Materials Chemistry C*.

[33] Wang B., Wu Q., Zhang Y., Ma L., Wang J. (2019). Auxetic B4N monolayer: a promising 2D material with in-plane negative Poisson’s ratio and large anisotropic mechanics. *ACS Applied Materials & Interfaces*.

[34] Peng R., Ma Y., He Z., Huang B., Kou L., Dai Y. (2019). Single-layer Ag2S: a two-dimensional bidirectional auxetic semiconductor. *Nano Letters*.

[35] Park C.-M., Sohn H.-J. (2007). Black phosphorus and its composite for lithium rechargeable batteries. *Advanced Materials*.

[36] Gopinadhan K., Hu S., Esfandiar A. (2019). Complete steric exclusion of ions and proton transport through confined monolayer water. *Science*.

[37] Li X., Huang C., Hu S. (2020). Negative and near-zero Poisson's ratios in 2D graphene/MoS2and graphene/h-BN heterostructures. *Journal of Materials Chemistry C*.

[38] Quhe R., Zheng J., Luo G. (2012). Tunable and sizable band gap of single-layer graphene sandwiched between hexagonal boron nitride. *NPG Asia Materials*.

[39] Lee C., Wei X., Kysar J. W., Hone J. (2008). Measurement of the elastic properties and intrinsic strength of monolayer graphene. *Science*.

[40] Kim K., Lambrecht W. R. L., Segall B. (1996). Elastic constants and related properties of tetrahedrally bonded BN, AlN, GaN, and InN. *Physical Review B*.

[41] Brugger K. (1965). Pure modes for elastic waves in crystals. *Journal of Applied Physics*.

[42] Hu S., Alsubaie A., Wang Y. (2017). Poisson's ratio of BiFeO_3_ thin films: X-ray reciprocal space mapping under variable uniaxial strain. *physica status solidi (a)*.

[43] Hamaker H. C. (1937). The London--van der Waals attraction between spherical particles. *Physica*.

[44] Gritsenko O. V., Schipper P. R. T., Baerends E. J. (1998). Effect of Pauli repulsion on the molecular exchange-correlation Kohn-sham potential: a comparative calculation of Ne_2_ and N_2_. *Physical Review A*.

[45] Gong Z., Shi X., Li J. (2020). Theoretical prediction of low-energy Stone-Wales graphene with an intrinsic type-III Dirac cone. *Physical Review B*.

[46] Froyen S., Harrison W. A. (1979). Elementary prediction of linear combination of atomic orbitals matrix elements. *Physical Review B*.

[47] Hu L., Zhao J., Yang J. (2015). Nano-scale displacement sensing based on van der Waals interactions. *Nanoscale*.

[48] Xie S., Tu L., Han Y. (2018). Coherent, atomically thin transition-metal dichalcogenide superlattices with engineered strain. *Science*.

[49] Geim A. K., Grigorieva I. V. (2013). Van der Waals heterostructures. *Nature*.

[50] Kresse G., Furthmüller J. (1996). Efficient iterative schemes forab initiototal-energy calculations using a plane-wave basis set. *Physical Review B*.

[51] Perdew J. P., Chevary J. A., Vosko S. H. (1992). Atoms, molecules, solids, and surfaces: applications of the generalized gradient approximation for exchange and correlation. *Physical Review B*.

[52] Grimme S. (2006). Semiempirical GGA-type density functional constructed with a long-range dispersion correction. *Journal of Computational Chemistry*.

[53] Grimme S., Antony J., Ehrlich S., Krieg H. (2010). A consistent and accurate ab initio parametrization of density functional dispersion correction (DFT-D) for the 94 elements H-Pu. *The Journal of Chemical Physics*.

[54] Bučko T., Lebègue S., Gould T., Ángyán J. G. (2016). Many-body dispersion corrections for periodic systems: an efficient reciprocal space implementation. *Journal of Physics: Condensed Matter*.

[55] Thonhauser T., Cooper V. R., Li S., Puzder A., Hyldgaard P., Langreth D. C. (2007). Van der Waals density functional: self-consistent potential and the nature of the van der Waals bond. *Physical Review B*.

[56] Heyd J., Scuseria G. E., Ernzerhof M. (2003). Hybrid functionals based on a screened coulomb potential. *The Journal of Chemical Physics*.

[57] Thurston R. N., Brugger K. (1964). Third-order elastic constants and the velocity of small amplitude elastic waves in homogeneously stressed media. *Physical Review*.

[58] Zhao J., Winey J. M., Gupta Y. M. (2007). First-principles calculations of second- and third-order elastic constants for single crystals of arbitrary symmetry. *Physical Review B*.

