# Supplementary material for: Tunable Negative Poisson's Ratio in Van der Waals Superlattice
Source: Research (Wash D C). 2021 Apr 10;2021:1904839. doi: 10.34133/2021/1904839 (PMC8054987; doi:10.34133/2021/1904839)
Supplement: Supplementary Materials — 1: interlayer binding energies and equilibrium distances results-Supplementary Table S1 2: elastic constants results-Supplementary Table S2 3: volume modulus and shear modulus results-Supplementary Table S3 4: interlayer binding energy-Supplementary Table S4 5: relationship between pz orbitals and NPR-Supplementary Figure S1 6: charge density-Supplementary Figure S2, Table S5 7: charge density. Table 8: the relationship between Poisson's ratio and stiffness. The relationship between θ and ν13 9. The relationship between electronic band structures and NPR-Supplementary Table S6. [file 1904839.f1.docx]

# Supplementary Information

# Tunable negative Poisson's ratio in van der Waals superlattice

Xiaowen Li^1^, Xiaobin Qiang^1^, Zhenhao Gong^1^, Yubo Zhang^1^, Penglai Gong^1^ and Lang Chen^*,1^

^1^Department of Physics, Southern University of Science and Technology, Shenzhen, Guangdong 518055, China.

*Correspondence and requests for materials should be addressed to L.C. (email: chenlang@sustech.edu.cn).

**Supplementary Information Outline:**

1. Interlayer binding energies and equilibrium distances results - Supplementary Table S1

2. Elastic constants results - Supplementary Table S2

3. Volume modulus and shear modulus results - Supplementary Table S3

4. Interlayer binding energy - Supplementary Table S4

5. Relationship between *p*_z_ orbitals and NPR - Supplementary Figure S1

6. Charge density - Supplementary Figure S2, Table S5

7. The relationship between Poisson’s ratio and stiffness

8. The relationship between θ and ν_13_

9. The relationship between electronic band structures and NPR- Supplementary Table S6

# 1. Interlayer binding energies and equilibrium distances results

**Table S1.** Interlayer binding energies (*E_bind_*) and equilibrium distances (*d*) for G/*h*-BN superlattice with different stacking modes.

| G/*h*-BN superlattice |  | |  | |  | |
| --- | --- | --- | --- | --- | --- | --- |
|  | *E_bind_*  (meV/Å^2^) | *d* (Å) | *E_bind_*  (meV/Å^2^) | *d* (Å) | *E_bind_*  (meV/Å^2^) | *d* (Å) |
| D2 | 39.72 | 3.345 | 31.86 | 3.270 | 41.08 | 3.108 |
| D3 | 36.44 | 3.520 | 36.97 | 3.481 | 40.96 | 3.356 |
| OptB88 | 43.13 | 3.448 | 44.34 | 3.416 | 50.16 | 3.274 |
| MBD | 36.36 | 3.509 | 37.49 | 3.470 | 42.70 | 3.313 |

# 2. Elastic constants results

**Table S2**. Computational results of elastic constants for G/*h*-BN, graphene and *h*-BN by density functional theory with different vdW corrections.

|  | | Method | C_11_ | C_12_ | C_13_ | C_33_ | C_44_ |
| --- | --- | --- | --- | --- | --- | --- | --- |
| G/*h*-BN | Stacking mode A | D2 | 948.7 | 185.6 | -7.8 | 40.9 | -2.3 |
|  |  | D3 | 908.7 | 184.9 | -8.5 | 23.3 | -5.7 |
|  |  | OptB88 | 924.4 | 185.0 | -6.1 | 45.0 | -1.3 |
|  |  | MBD | 909.8 | 180.2 | -8.6 | 24.1 | -4.5 |
|  | Stacking mode B | D2 | 970.2 | 189.7 | -8.6 | 51.2 | -1.1 |
|  |  | D3 | 921.6 | 188.3 | -6.9 | 29.5 | -4.3 |
|  |  | OptB88 | 933.8 | 185.4 | -6.3 | 45.7 | -2.8 |
|  |  | MBD | 920.9 | 182.5 | -8.2 | 34.4 | -1.3 |
|  | Stacking mode C | D2 | 1022.9 | 203.4 | -6.8 | 62.9 | 12.6 |
|  |  | D3 | 961.1 | 201.2 | -1.2 | 38.4 | 7.5 |
|  |  | OptB88 | 971.2 | 193.0 | -1.4 | 49.0 | 8.7 |
|  |  | MBD | 963.5 | 190.4 | -5.6 | 30.5 | 6.1 |
| Monolayer | Graphene | Expt.[1] | 349.5±51.4 | 57.7±8.5 |  | | |
|  |  | D2 | 346.2 | 56.4 |  |  |  |
|  |  | D3 | 349.8 | 58.4 |  |  |  |
|  |  | OptB88 | 348.8 | 55.5 |  |  |  |
|  |  | MBD | 351.9 | 59.1 |  |  |  |
|  | *h*-BN | Expt.[2] | 283.2 | 53.8 |  |  |  |
|  |  | D2 | 284.1 | 48.0 |  |  |  |
|  |  | D3 | 270.4 | 53.0 |  |  |  |
|  |  | OptB88 | 287.4 | 56.4 |  |  |  |
|  |  | MBD | 279.9 | 55.1 |  |  |  |
| ^a^For monolayer materials, the unit of elastic constants is Nm^-1^. For G/*h*-BN superlattices, the unit of elastic constants is GPa. | | | | | | | |

# 3. Volume modulus and shear modulus results

For Voigt model, bulk modulus $K_{V}$ and shear modulus $G_{V}$ are expressed as:

$$\begin{aligned} \left\{ \begin{aligned} 9K_{V}=\left( C_{11}+C_{22}+C_{33} \right)+2\left( C_{12}+C_{23}+C_{31} \right) \\ 15G_{V}=\left( C_{11}+C_{22}+C_{33} \right)-\left( C_{12}+C_{23}+C_{31} \right)+3\left( C_{44}+C_{55}+C_{66} \right) \end{aligned} \right.\# \end{aligned}$$

For Reuss model, bulk modulus $K_{R}$ and shear modulus $G_{R}$ are expressed as:

$$\begin{aligned} \left\{ \begin{aligned} \frac{1}{K_{R}}=\left( S_{11}+S_{22}+S_{33} \right)+2\left( S_{12}+S_{23}+S_{31} \right) \\ \frac{15}{G_{R}}=4\left( S_{11}+S_{22}+S_{33} \right)-4\left( S_{12}+S_{23}+S_{31} \right)+3\left( S_{44}+S_{55}+S_{66} \right) \end{aligned} \right.\# \end{aligned}$$

For hexagonal crystals, the in-plane mechanical properties of G/*h*-BN superlattice are isotropic (*Y*_11_= *Y*_22_, *v*_12_= *v*_21_, *v*_13_= *v*_23_), and there are only five independent elastic constants (*C*_11_, *C*_12_, *C*_13_, *C*_33_ and *C*_44_). Therefore, we can get:

$$\begin{aligned} \left\{ \begin{aligned} K_{V}=\frac{1}{9}\left( 2C_{11}+C_{33}+2C_{12}+4C_{13} \right) \\ G_{V}=\frac{1}{30}\left( 7C_{11}+2C_{33}-5C_{12}-4C_{13}+12C_{44} \right) \end{aligned} \right.\# \end{aligned}$$

$$\begin{aligned} \left\{ \begin{aligned} K_{R}=\frac{1}{2S_{11}+S_{33}+2S_{12}+4S_{13}} \\ G_{R}=\frac{15}{14S_{11}+4S_{33}-10S_{12}-8S_{13}+6S_{44}} \end{aligned} \right.\# \end{aligned}$$

Under the Voigt-Reuss-Hill average approximation, the bulk modulus *K_VRH_* and shear modulus *G_VRH_* are expressed as:

$$\begin{aligned} \left\{ \begin{aligned} K_{VRH}=\frac{K_{V}+K_{R}}{2} \\ G_{VRH}=\frac{G_{V}+G_{R}}{2} \end{aligned} \right.\# \end{aligned}$$

**Table S3**. Computational results of volume modulus and shear modulus for G/*h*-BN by density functional theory with optB88 vdW corrections.

|  | | K_V_ | G_V_ | K_R_ | G_R_ | B_VRH_ | G_VRH_ |
| --- | --- | --- | --- | --- | --- | --- | --- |
| G/*h*-BN | Stacking mode A | 248.8 | 188.2 | 40.7 | -0.2 | 144.8 | 94.0 |
|  | Stacking mode B | 251.0 | 189.8 | 41.3 | -0.5 | 146.2 | 94.6 |
|  | Stacking mode C | 263.5 | 201.4 | 45.0 | 1.3 | 154.3 | 101.3 |

# 4. Interlayer binding energy

**Table S4**. Best-Fit Parameters for the *E_bind_* of G/*h*-BN superlattices in stacking mode A, B and C under $\text{ε}_{\text{x}}$=0 and $\text{ε}_{\text{x}}$=-0.08.

|  | $\varepsilon_{x}$=0.00 | | | $\varepsilon_{x}$=0.08 | | |
| --- | --- | --- | --- | --- | --- | --- |
|  | $\varepsilon$ (meV) | $\rho_{1}$ (Å^-2^) | $\sigma$ (Å) | $\varepsilon'$ (meV) | $\rho_{1}'$ (Å^-2^) | $\sigma'$(Å) |
| Stacking mode A | 6.909 | 0.373 | 3.448 | 7.354 | 0.351 | 3.478 |
| Stacking mode B | 7.238 | 0.373 | 3.416 | 7.712 | 0.351 | 3.446 |
| Stacking mode C | 8.917 | 0.373 | 3.274 | 9.959 | 0.351 | 3.280 |

# 5. Relationship between *p_z_* orbitals and NPR.

Figure S1. The basis vector of (**a**) graphene lattice and (**b**) reciprocal lattice in k-space. The red and green arrows represent basis vectors, and black arrows represent connection vectors.

The basis vectors of graphene lattice are

$$a_{1}=\frac{3a}{2}i+\frac{\sqrt{3}a}{2}j$$

$$a_{2}=\frac{3a}{2}i-\frac{\sqrt{3}a}{2}j$$

The basis vectors of reciprocal lattice in k space are

$$b_{1}=\frac{2\pi}{3a}i+\frac{2\sqrt{3}\pi}{3a}j$$

$$b_{2}=\frac{2\pi}{3a}i-\frac{2\sqrt{3}\pi}{3a}j$$

For each carbon atom, there are three nearest neighbors, and the connection vectors between them are

$$\Delta_{1}=\frac{a}{2}i+\frac{\sqrt{3}a}{2}j$$

$$\Delta_{2}=\frac{a}{2}i-\frac{\sqrt{3}a}{2}j$$

$$\Delta_{3}=-ai$$

The result of normalized wave functions under tight binding approximation are as follows:

$$\phi_{A}\left( k,r \right)=\frac{1}{\sqrt{N}}\sum_{m} e^{ik\cdot R_{m}^{A}}\varphi\left( r-R_{m}^{A} \right)$$

$$\phi_{B}\left( k,r \right)=\frac{1}{\sqrt{N}}\sum_{m} e^{ik\cdot R_{m}^{B}}\varphi\left( r-R_{m}^{B} \right)$$

The wave function of graphene can be obtained by linear combination of atomic orbitals (LCAO):

$$\psi_{p_{z}}\left( k,r,\delta\right)=C\left( \phi_{A}\left( k,r \right)+{e^{-ik\cdot\delta}\phi}_{B}\left( k,r \right) \right)$$

Therefore, the length of *p_z_* orbitals is

$$L_{z}\left( k,\delta\right)=\left\langle\psi_{p_{z}}\left( k,\delta\right) | \left| z \right| | \psi_{p_{z}}\left( k,\delta\right) \right\rangle$$

$$\begin{aligned} =\frac{\left\langle\phi_{A}\left( k \right) | \left| z \right| | \phi_{A}\left( k \right) \right\rangle+e^{-ik\cdot\delta}\left\langle\phi_{A}\left( k \right) | \left| z \right| | \phi_{B}\left( k \right) \right\rangle+e^{ik\cdot\delta}\left\langle\phi_{B}\left( k \right) | \left| z \right| | \phi_{A}\left( k \right) \right\rangle+\left\langle\phi_{B}\left( k \right) | \left| z \right| | \phi_{B}\left( k \right) \right\rangle}{\left\langle\phi_{A}\left( k \right) | \phi_{A}\left( k \right) \right\rangle+e^{-ik\cdot\delta}\left\langle\phi_{A}\left( k \right) | \phi_{B}\left( k \right) \right\rangle+e^{ik\cdot\delta}\left\langle\phi_{B}\left( k \right) | \phi_{A}\left( k \right) \right\rangle+\left\langle\phi_{B}\left( k \right) | \phi_{B}\left( k \right) \right\rangle}\# \end{aligned}$$

where

$$\left\langle\phi_{A}\left( k \right) | \left| z \right| | \phi_{A}\left( k \right) \right\rangle=\left\langle\frac{1}{\sqrt{N}}\sum_{m} e^{ik\cdot R_{m}^{A}}\varphi\left( r-R_{m}^{A} \right) | \left| z \right| | \frac{1}{\sqrt{N}}\sum_{m} e^{ik\cdot R_{m}^{A}}\varphi\left( r-R_{m}^{A} \right) \right\rangle$$

$$=\frac{1}{N}\sum_{m} \left\langle\varphi\left( r-R_{m}^{A} \right) | \left| z \right| | \varphi\left( r-R_{m}^{A} \right) \right\rangle$$

$$=\left\langle\varphi\left( r-R_{m}^{A} \right) | \left| z \right| | \varphi\left( r-R_{m}^{A} \right) \right\rangle$$

$$=l_{p_{z}}$$

$$\left\langle\phi_{A}\left( k \right) | \left| z \right| | \phi_{B}\left( k \right) \right\rangle=\left\langle\frac{1}{\sqrt{N}}\sum_{m} e^{ik\cdot R_{m}^{A}}\varphi\left( r-R_{m}^{A} \right) | \left| z \right| | \frac{1}{\sqrt{N}}\sum_{m} e^{ik\cdot R_{m}^{B}}\varphi\left( r-R_{m}^{B} \right) \right\rangle$$

$$=\frac{1}{N}\sum_{m} e^{ik\cdot\left( R_{m}^{B}-R_{m}^{A} \right)}\left\langle\varphi\left( r-R_{m}^{A} \right) | \left| z \right| | \varphi\left( r-R_{m}^{A} \right) \right\rangle$$

$$\approx\frac{1}{3}\left( e^{ik\cdot\Delta_{1}}+e^{ik\cdot\Delta_{2}}+e^{ik\cdot\Delta_{3}} \right)\left\langle\varphi\left( r-R_{m}^{A} \right) | \left| z \right| | \varphi\left( r-R_{m}^{A} \right) \right\rangle$$

$$=\frac{1}{3}\left( 2e^{ik_{x}\frac{a}{2}}\cos\left( k_{y}\frac{\sqrt{3}a}{2} \right)+e^{-ik_{x}a} \right)\left\langle\varphi\left( r-R_{m}^{A} \right) | \left| z \right| | \varphi\left( r-R_{m}^{A} \right) \right\rangle$$

$$=\frac{1}{3}\left( 2e^{ik_{x}\frac{a}{2}}\cos\left( k_{y}\frac{\sqrt{3}a}{2} \right)+e^{-ik_{x}a} \right)l_{p_{z}}$$

$$\left\langle\phi_{A}\left( k \right) | \phi_{B}\left( k \right) \right\rangle=\left\langle\frac{1}{\sqrt{N}}\sum_{m} e^{ik\cdot R_{m}^{A}}\varphi\left( r-R_{m}^{A} \right) | \frac{1}{\sqrt{N}}\sum_{m} e^{ik\cdot R_{m}^{B}}\varphi\left( r-R_{m}^{B} \right) \right\rangle$$

$$=\frac{1}{N}\sum_{m} e^{ik\cdot\left( R_{m}^{B}-R_{m}^{A} \right)}\left\langle\varphi\left( r-R_{m}^{A} \right) | \varphi\left( r-R_{m}^{B} \right) \right\rangle$$

$$\approx\delta_{A,B}$$

Therefore, the length ($L_{z}$) of *p*_z_ electrons with all momentums should be the integral of $l_{z}\left( k \right)$ in the first Brillouin zone (BZ) :

$$L_{z}\left( \delta\right)=\frac{1}{S_{BZ}}\iint_{S_{BZ}} l_{z}\left( k \right){dk}^{2}$$

$$=\frac{1}{S_{BZ}}\iint_{S_{BZ}} \left[ l_{p_{z}}+\frac{1}{3}\left( 2e^{ik_{x}\frac{a}{2}}\cos\left( k_{y}\frac{\sqrt{3}a}{2} \right)+e^{-ik_{x}a} \right)l_{p_{z}}\cos\left( k\cdot\delta\right) \right]{dk}^{2}$$

$$=l_{p_{z}}\left( 1+\frac{l_{AB}}{{3S}_{BZ}}\iint_{S_{BZ}} \left( 2e^{ik_{x}\frac{a}{2}}\cos\left( k_{y}\frac{\sqrt{3}a}{2} \right)+e^{-ik_{x}a} \right)\cos\left( k\cdot\delta\right){dk}^{2} \right)$$

$$=l_{p_{z}}f\left( \delta\right)$$

where

$$f\left( \delta\right)=1+\frac{1}{{3S}_{BZ}}\iint_{S_{BZ}} \left( 2e^{ik_{x}\frac{a}{2}}\cos\left( k_{y}\frac{\sqrt{3}a}{2} \right)+e^{-ik_{x}a} \right)\cos\left( k\cdot\delta\right){dk}^{2}$$

# 6. Charge density


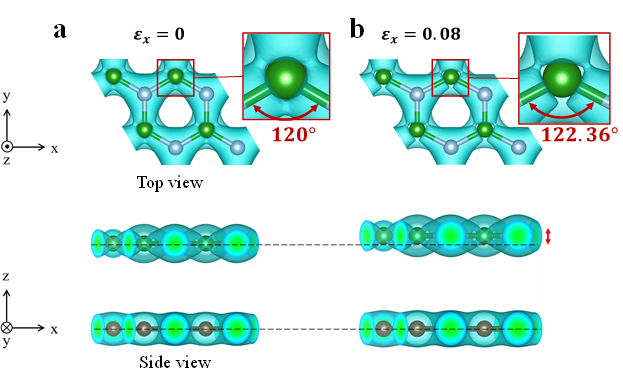


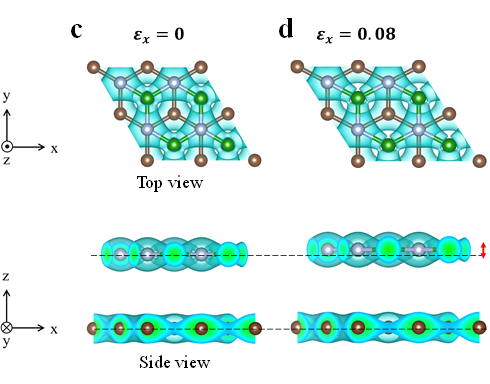


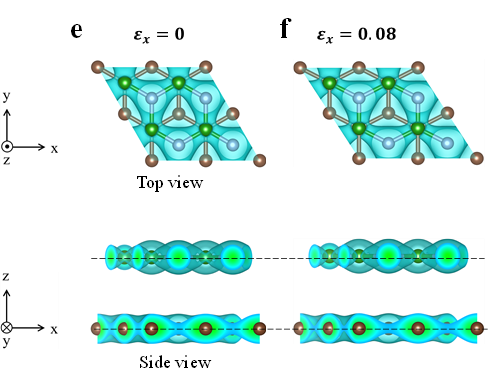


**Figure S2**. Front and side views of the stacking mode (**a**, **b**) A, (**c**, **d**) B and (**e**, **f**) C of the charge density with the isosurface of 0.103 Å^−3^ under in-plane strain $\text{ε}_{\text{x}}$=0 and $\text{ε}_{\text{x}}$=0.08.

**Table S5**. The length of *p*_z_ orbital in each layer of G/*h*-BN superlattice.

|  |  | $L_{Z}$ (Å) | ${\delta L}_{Z}/L_{Z}$ |
| --- | --- | --- | --- |
| Stacking mode A | Graphene | 0.654 | 2.3% |
|  | *h*-BN | 0.775 | 1.8% |
| Stacking mode B | Graphene | 0.657 | 2.3% |
|  | *h*-BN | 0.780 | 1.8% |
| Stacking mode C | Graphene | 0.683 | 2.4% |
|  | *h*-BN | 0.818 | 1.9% |
| ^a^At the equilibrium position $\varepsilon_{x}=0$, the length of *p*_z_ orbital is $L_{Z}$; under the in-plane strain $\varepsilon_{x}$ = 0.08, the length of *p*_z_ orbital is $L_{Z}+{\delta L}_{Z}$. | | | |

# 7. The relationship between Poisson’s ratio and stiffness

The stiffness tensor of a hexagonal crystal system can be written as follows:

$C_{\alpha\beta}=\left( \begin{matrix} C_{11} & C_{12} & C_{13} & 0 & 0 & 0 \\ C_{12} & C_{11} & C_{13} & 0 & 0 & 0 \\ C_{13} & C_{13} & C_{33} & 0 & 0 & 0 \\ 0 & 0 & 0 & C_{44} & 0 & 0 \\ 0 & 0 & 0 & 0 & C_{44} & 0 \\ 0 & 0 & 0 & 0 & 0 & \frac{C_{11}-C_{12}}{2} \end{matrix} \right)$.

The compliance tensor can be obtained by taking inverse of the stiffness tensor:

$S_{\alpha\beta}=C_{\alpha\beta}^{-1}=\left( \begin{matrix} S_{11} & S_{12} & S_{13} & 0 & 0 & 0 \\ S_{12} & S_{11} & S_{13} & 0 & 0 & 0 \\ S_{13} & S_{13} & S_{33} & 0 & 0 & 0 \\ 0 & 0 & 0 & S_{44} & 0 & 0 \\ 0 & 0 & 0 & 0 & S_{44} & 0 \\ 0 & 0 & 0 & 0 & 0 & 2\left( S_{11}-S_{12} \right) \end{matrix} \right)$.

The in-plane Poisson’s ratio v_12_ and the out-of-plane Poisson’s ratio v_13_:

$\left\{ \begin{aligned} v_{12}=-\frac{S_{12}}{S_{11}} \\ v_{13}=-\frac{S_{13}}{S_{11}} \end{aligned} \right.$.

After simplification,

$\left\{ \begin{aligned} v_{12}=\frac{{C_{13}}^{2}-C_{12}C_{33}}{{C_{13}}^{2}-C_{11}C_{33}} \\ v_{13}=\frac{C_{13}\left( C_{12}-C_{11} \right)}{{C_{13}}^{2}-C_{11}C_{33}} \end{aligned} \right.$.

Therefore, we can verify the accuracy of the results by the relationship between Poisson’s ratio and stiffness tensor.

# 8. The relationship between θ and ν_13_

When i fixed in the x direction and *j* varying in the y-z plane by an angle of θ, the transformation from the stress *σ_β_* (unprimed) to the stress *σ_β’_* in arbitrary system (primed) is described by:

$\underset{\sigma_{\beta'}}{\underbrace{\left( \begin{aligned} \sigma_{1'} \\ \sigma_{2'} \\ \sigma_{3'} \\ \sigma_{4'} \\ \sigma_{5'} \\ \sigma_{6'} \end{aligned} \right)}}=\underset{T_{\sigma_{\beta'\beta}}}{\underbrace{\left( \begin{matrix} 1 & 0 & 0 & 0 & 0 & 0 \\ 0 & \cos^{2} \theta& \sin^{2} \theta& 2\sin\theta\cos\theta& 0 & 0 \\ 0 & \sin^{2} \theta& \cos^{2} \theta& -2sin \theta\cos\theta& 0 & 0 \\ 0 & -sin \theta\cos\theta& \sin\theta\cos\theta& \cos^{2} \theta-\sin^{2} \theta& 0 & 0 \\ 0 & 0 & 0 & 0 & \cos\theta& -\sin\theta\\ 0 & 0 & 0 & 0 & \sin\theta& \cos\theta\end{matrix} \right)}}\underset{\sigma_{\beta}}{\underbrace{\left( \begin{aligned} \sigma_{1} \\ \sigma_{2} \\ \sigma_{3} \\ \sigma_{4} \\ \sigma_{5} \\ \sigma_{6} \end{aligned} \right)}}$.

Note that we use contracted notations (11→1, 22→2, 33→3, 13→4, 23→5,12→3, *C_ijkl_*→*C_αβ_, C_ijklmn_*→*C_αβγ_*) for the tensor indices. The transformation from the strain *ε_α_* (unprimed) to the strain *ε_α’_* in arbitrary system (primed) is described by:

$\underset{\varepsilon_{\alpha'}}{\underbrace{\left( \begin{aligned} \varepsilon_{1'} \\ \varepsilon_{2'} \\ \varepsilon_{3'} \\ \varepsilon_{4'} \\ \varepsilon_{5'} \\ \varepsilon_{6'} \end{aligned} \right)}}=\underset{T_{\varepsilon_{\alpha'\alpha}}}{\underbrace{\left( \begin{matrix} 1 & 0 & 0 & 0 & 0 & 0 \\ 0 & \cos^{2} \theta& \sin^{2} \theta& \sin\theta\cos\theta& 0 & 0 \\ 0 & \sin^{2} \theta& \cos^{2} \theta& -sin \theta\cos\theta& 0 & 0 \\ 0 & -2sin \theta\cos\theta& 2sin \theta\cos\theta& \cos^{2} \theta-\sin^{2} \theta& 0 & 0 \\ 0 & 0 & 0 & 0 & \cos\theta& -\sin\theta\\ 0 & 0 & 0 & 0 & \sin\theta& \cos\theta\end{matrix} \right)}}\underset{\varepsilon_{\alpha}}{\underbrace{\left( \begin{aligned} \varepsilon_{1} \\ \varepsilon_{2} \\ \varepsilon_{3} \\ \varepsilon_{4} \\ \varepsilon_{5} \\ \varepsilon_{6} \end{aligned} \right)}}$.

The compliance coefficients *S_α’β’_* are defined as the proportionality constants between stress *σ_β’_* and strain by the Hooke's law:

$\varepsilon_{\alpha'}=S_{\alpha'\beta'}\sigma_{\beta'}$.

It can be expressed by the coordinate transformation equation:

$T_{\varepsilon_{\alpha'\alpha}}\varepsilon_{\alpha}=S_{\alpha'\beta'}T_{\sigma_{\beta'\beta}}\sigma_{\beta}$.

We obtain

$\varepsilon_{\alpha}=\underset{S_{\alpha\beta}}{\underbrace{{{T_{\varepsilon_{\alpha'\alpha}}}^{-1}S}_{\alpha'\beta'}T_{\sigma_{\beta'\beta}}}}\sigma_{\beta}$,

and

$S_{\alpha'\beta'}={T_{\varepsilon_{\alpha'\alpha}}S}_{\alpha\beta}{T_{\sigma_{\beta'\beta}}}^{-1}$.

The out-of-plane Poisson’s ratio *v*_13_:

$v_{13}=-\frac{S_{13}}{S_{11}}$.

After simplification,

$v_{13}(\theta)=-\frac{\left( C_{11}-C_{12} \right)C_{13}\cos^{2} \theta-\left( {C_{13}}^{2}-C_{12}C_{13} \right)\sin^{2} \theta}{{C_{13}}^{2}-C_{11}C_{13}}$.

# 9. The relationship between electronic band structures and NPR

Hamiltonian matrix elements are

$$\begin{aligned} \left\{ \begin{aligned} H_{11}=E_{C1pz}+f_{CC\pi}\left( {r_{11}}^{1} \right)\sum_{i} e^{-k\cdot{R_{11}}^{i}} \\ H_{22}=E_{C2pz}+f_{CC\pi}\left( {r_{11}}^{1} \right)\sum_{i} e^{-k\cdot{R_{11}}^{i}} \\ H_{33}=E_{Npz}+f_{NN\pi}\left( {r_{11}}^{1} \right)\sum_{i} e^{-k\cdot{R_{11}}^{i}} \\ H_{12}=f_{CC\pi}\left( {r_{12}}^{1} \right)\sum_{i} e^{-k\cdot{R_{12}}^{i}}+f_{CC\pi}\left( {r_{12}}^{2} \right)\sum_{i} e^{-k\cdot{R_{12}}^{i}} \\ H_{13}=\left[ {-cos}^{2} \theta f_{CN\sigma}\left( {r_{13}}^{1} \right)+{sin}^{2} \theta f_{CN\pi}\left( {r_{13}}^{1} \right) \right]\left[ e^{-k\cdot{R_{13}}^{1}}+e^{-k\cdot{R_{13}}^{2}}+e^{-k\cdot{R_{13}}^{3}} \right] \\ H_{23}=f_{CN\sigma}\left( {r_{23}}^{1} \right)e^{-k\cdot{R_{23}}^{1}} \end{aligned} \right.\# \end{aligned}$$

Similarly, the overlap integral matrix elements are

$$\begin{aligned} \left\{ \begin{aligned} S_{11}=1+g_{CC\pi}\left( {r_{11}}^{1} \right)\sum_{i} e^{-k\cdot{R_{11}}^{i}} \\ S_{22}=1+g_{CC\pi}\left( {r_{11}}^{1} \right)\sum_{i} e^{-k\cdot{R_{11}}^{i}} \\ S_{33}=1+g_{NN\pi}\left( {r_{11}}^{1} \right)\sum_{i} e^{-k\cdot{R_{11}}^{i}} \\ S_{12}=g_{CC\pi}\left( {r_{12}}^{1} \right)\sum_{i} e^{-k\cdot{R_{12}}^{i}}+g_{CC\pi}\left( {r_{12}}^{2} \right)\sum_{i} e^{-k\cdot{R_{12}}^{i}} \\ S_{13}=\left[ {-cos}^{2} \theta g_{CN\sigma}\left( {r_{13}}^{1} \right)+{sin}^{2} \theta g_{CN\pi}\left( {r_{13}}^{1} \right) \right]\left[ e^{-k\cdot{R_{13}}^{1}}+e^{-k\cdot{R_{13}}^{2}}+e^{-k\cdot{R_{13}}^{3}} \right] \\ S_{23}=g_{CN\sigma}\left( {r_{23}}^{1} \right)e^{-k\cdot{R_{23}}^{1}} \end{aligned} \right.\# \end{aligned}$$

where $E_{ipz}$ represents the lattice energy of the *i*th atom, and ${r_{\mathrm{ij}}}^{1}$ and ${R_{\mathrm{ij}}}^{i}$ represent the distance and distance vector between the *i*th and *j*th atoms, respectively. The distance-dependent overlap integral is determined by the formula

$$\begin{aligned} g_{ij\sigma}\left( d_{ij} \right)=S_{ij\sigma}e^{p_{ij}\left( 1-\frac{d_{ij}}{d_{0}} \right)}\#\left( 10 \right) \end{aligned}$$

Here, *d*_0_ represents the interfacial layer equilibrium distance, and *S_ijσ_* is the overlap integral between *p*_z_ orbitals at *d*_0_. *d_ij_* is the distance between the *i*th and *j*th atoms, and *p_ij_* is the decay constant for the integral[3].

**Table S6**. The values of *V_ijσ_*, *q_ij_*, *S_ijσ_* and *p_ij_*. The unit is eV.

| $E_{C1pz}$ | | 0.4 |
| --- | --- | --- |
| $E_{C2pz}$ | | 0.4 |
| $E_{Npz}$ | | 1.8 |
| $f_{CC\pi}$ | $V_{CC\pi}$ | -1.753 |
|  | $q_{CC}$ | 1.274 |
| $g_{CC\pi}$ | $S_{CC\pi}$ | 0.4767 |
|  | $p_{CC}$ | 1.175 |
| $f_{NN\pi}$ | $V_{NN\pi}$ | -1.1 |
|  | $q_{NN}$ | 0 |
| $g_{NN\pi}$ | $S_{NN\pi}$ | 0.12 |
|  | $p_{NN}$ | 0 |
| $f_{CN\pi}$ | $V_{CN\pi}$ | -2 |
|  | $q_{CN}$ | 0 |
| $g_{CN\pi}$ | $S_{CN\pi}$ | 0.25 |
|  | $p_{CN}$ | 0 |
| $f_{CN\sigma}$ | $V_{CN\sigma}$ | -0.31 |
|  | $q_{CN}$ | 2.51 |
| $g_{CN\sigma}$ | $S_{CN\sigma}$ | 0.05 |
|  | $p_{CN}$ | 0 |

# Reference

[1] C. Lee, X. Wei, J. W. Kysar *et al.*, "Measurement of the Elastic Properties and Intrinsic Strength of Monolayer Graphene," *Science*, vol. 321, no. 5887, pp. 385-388, 2008.

[2] K. Kim, W. R. L. Lambrecht and B. Segall, "Elastic constants and related properties of tetrahedrally bonded BN, AlN, GaN, and InN," *Physical Review B*, vol. 53, no. 24, pp. 16310-16326, 1996.

[3] Z. Gong, X. Shi, J. Li *et al.*, "Theoretical prediction of low-energy Stone-Wales graphene with an intrinsic type-III Dirac cone," *Physical Review B*, vol. 101, no. 15, pp. 155427, 2020.
